# Views on artificial intelligence (AI) assisted clinical trials

**DOI:** 10.6026/97320630017616

**Published:** 2021-06-30

**Authors:** Arthi Balasundaram, C Stalin, Mohan Krishna Ghanta

**Affiliations:** 1 Biohymns Innovations Pte ltd, Singapore; 2 Pharmacovigilance Associate, Pharmacovigilance Programme of India (PVPI), Indian Pharmacopoeia Commission, Ghaziabad, India-201002; 3 Department of Pharmacology, MVJ Medical College and Research Hospital, Hoskote, Bangalore-562114, Karnataka, India

**Keywords:** Artificial intelligence, questionnaire study, clinical trials

## Abstract

It is of interest to document the views of medical professionals on the application of artificial intelligence (using known data for the prediction of unknown events) in clinical trials using a web survery with a structured questionnaire from 377 subjects. The questionnaire contained 17 statements which were categorised into awareness (1,2 statements), perception (3-10 statements) and opinion (11-17 statements). The data obtained was compared between the subjects using two tailed Fisher's exact test with p-value <0.05 for data significance analysis. Data shows that majority of professionals have possitive views on the application of artificial intelligence in clinical trials. This will accelarrate the drug evaluation process. However, the use of emerging tools such as AI will not replace human subjects in this context.

## Background

New drugs require more than 10 years to reach the market [1]. Hence, investment in drug design, research, development and formulation comes with high risk for pharma companies [2,3]. Therefore, the use of artificial intelligence help clinicians and researcher to identify specific targets in this context [4].

## Materials and Methods:

This study was conducted by BioHymns Innovations Pte. Ltd, Singapore, during Dec 2019 to May 2020 in Tamil Nadu, India. The sample size was measured using the Raosoft online calculator (Raosoft) and included 377 subjects. Medical doctors, who are involved or have been involved in clinical trials as investigator or co-investigator and medical Doctors who are interested in participating in this online survey were included in the study. Medical doctors who are not involved or have not been involved in clinical trials, non-medical doctors and medical doctors not interested in participating this online survey were excluded from the study.

## Study procedure:

This was a questionnaire-based study. Regarding questionnaire validity and reliability, a structured questionnaire was developed after a thorough literature review, which was conducted initially by the chief investigator and research papers were shortlisted for further discussion among the research team. All the views, thoughts and concerns on the proposed study were taken into consideration during the design phase. An initial draft of the questionnaire was designed after the research team had reviewed all the selected papers comprehensively. Individual survey items were reviewed by a group of medical professionals and consensus were reached regarding the clarity and importance of each item. The validation process was further expanded by piloting the questionnaire with four experienced doctors who meet the eligibility criteria and are not aware of this study. There was voluntary participation by the physicians. The questionnaire was framed in English. The questionnaire comprised 17 statements which were sectioned in to 3 events like awareness (1,2 statements), perception (3-10 statements) and opinion (11-17 statements). After obtaining ethics committee approval VISTASSPS/IEC/VIII/2019/04, the questionnaire was shared to professionals involved in clinical trials using survey Google forms.

## Statistical analysis:

An anonymous questionnaire was shared through Google forms to all participants. Basic statistics for the responses was done and represented as total number and percent. The data obtained was compared between the specialities using two tailed Fisher's exact test. P-value <0.05 was taken as significant.

## Results:

This study included 377 participants comprising resident doctors (N= 143), doctors working as clinical research associates (N= 12),paediatricians (N= 7), general physicians (N= 47), pharmacologists (N= 161), and clinical trial physicians (N= 7). The questionnaire consisting 17 statements have been categorized in to 3 types as for awareness, perception and opinion. The statements in the questionnaire were enlisted in Table 1(see PDF). The response rate towards the questionnaire statements was 100%. Responses against questionnaire statements The consolidated responses were tabulated in Table 1(see PDF). To describe the responses in general, majority (83.5 % & 65.5%) of the participants were aware of the AI based health care delivery and clinical trials. Most of the participants identified the potentiality of AI in, clinical trial processes, time saving or accelerating drug development, cost-effectiveness, and handling vast data. The AI based clinical trials was supported by large number of participants, but some has suggested that AI cannot substitute human intelligence and also, might raise ethical and legal concerns.

## Awareness:

**Figure 1:**
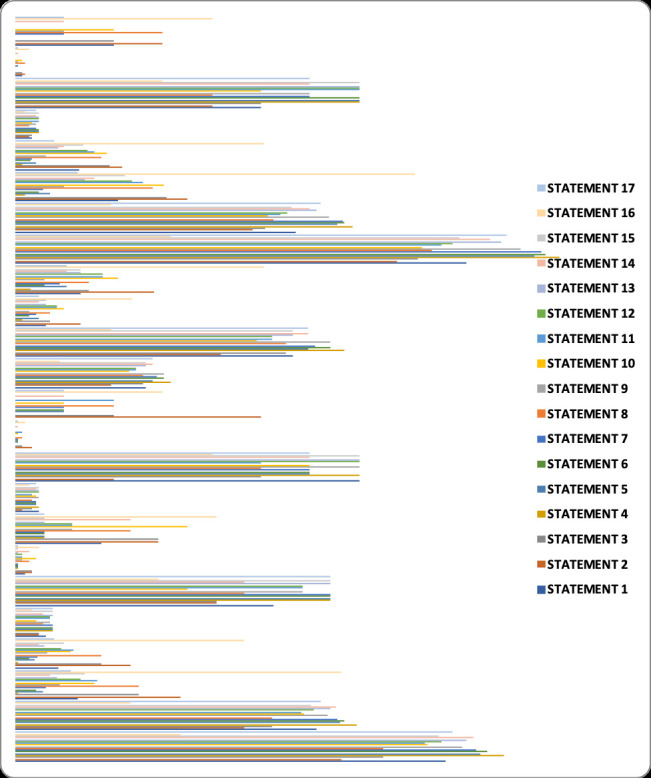
Questionnaire statement wise specialists' responses for AI based clinical trials. Y-positive response, N- negative response, %-percentage of response.

Statements 1 and 2 were categorised for analysis of awareness in this study. The responses were analysed as per the category of speciality. 220 positive responses out of 286 towards awareness were obtained from resident doctors. 16/24, 9/14, 66/94, 242/322, and 9/14 positive responses were obtained from clinical research associates, paediatricians, general physicians, pharmacologists, and clinical trial physicians respectively (Figure 1).

## Perception:

Statements from 3 to 10 comprised analysis for perception. 1012/1144, 75/96, 48/56, 319/376, 1119/1288, and 47/56 positive responses revealed the perception or recognition of the scope of AI in clinical trials by residents, clinical research associates, paediatricians, general physicians, pharmacologists, and clinical trial physicians respectively (Figure 1).

## Opinion

**Figure 2:**
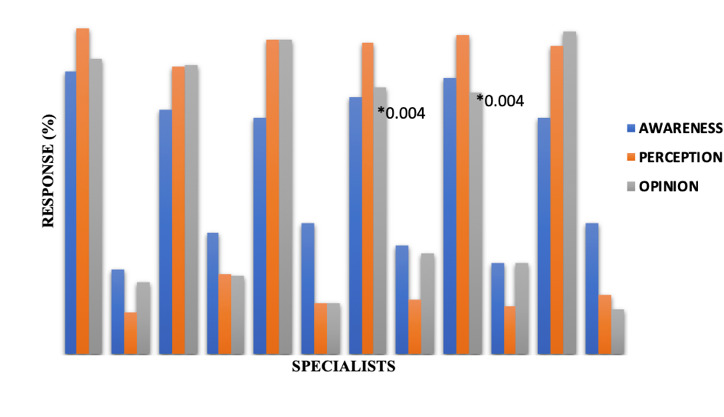
Awareness, perception and opinion responses of medical specialists for AI based clinical trials. CRA- Clinical Research Associate, PED- Paediatrician, GEN P- General Physician, PHARM- Pharmacologists, CTP- Clinical trial Physicians. *- p value obtained with two tailed Fisher's exact test.

Most of the specialists’ responses (residents- 805/1001, clinical research associates- 66/84, paediatricians- 42/49, general physicians- 239/329, pharmacologists- 847/1127, and clinical trial physicians- 43/49) have supported the AI in clinical trials [Figure 1]. But in comparison, the pharmacologists and general physicians' responses to opinion section showed significant difference (P value-0.004 and 0.004 respectively) to other groups (Figure 2).

## Discussion:

It is of interest to document the awareness, perception and opinion on AI based clinical trials amongst medical professionals using an online questionnaire survey. The study results statistically revealed comparable positive responses of awareness, perception and opinion between the specialities or specialists of medical profession. The pharmacologists and general physicians' responses in comparison to other specialities for opinion showed significant difference (P value-0.004 and 0.004 respectively) to other groups. This may be due to the responses to statement 16 where they suggested that AI cannot replace human intelligence. Majority of the medical specialities’ responses have shown that AI can cause ethical and legal concerns in clinical trials. Most of the specialists (88.85%) have supporting opinion towards AI based clinical trials, for accelerating the clinical trial process, aiding in patient recruitment and patient monitoring with suggestion of its usage (83.55%). These processes in clinical trials are very important and may decide the clinical trial outcome. It is known that these processes like substandard patient recruitment and withholding, ineffective patient monitoring contribute to the failure of clinical trial or raise of trial costs [5]. The participants’ responses in this study state that AI cannot be a substitute of human intelligence. This is supported by other studies [6,7,8]. Conversely, another study suggested that replacement of doctors might happen based upon the rapid scientific advancements. IBM’s Watson database was developed comprising vast information of publications and medical records which may assist in accurate diagnosis and treatments. This database also has genome information which may aid in personalised medicine [9,10,11]. Majority of participants (82.2%) suggested that AI may increase the success rates of clinical trials. Several difficulties are confronted in use of AI for convalescing outputs in drug development and healthcare. Perception of the risks and difficulties engrossed in its use and operation is dominant for improvement of responsive directives in this area [12].

## Conclusion:

Data shows that the medical professionals have awareness, perception and opinion on AI based clinical trials for further consideration in this domain.
